# Food insecurity as a risk factor of sarcopenic obesity in older adults

**DOI:** 10.3389/fnut.2022.1040089

**Published:** 2022-10-20

**Authors:** Diana Fonseca-Pérez, Cecilia Arteaga-Pazmiño, Claudia P. Maza-Moscoso, Sara Flores-Madrid, Ludwig Álvarez-Córdova

**Affiliations:** ^1^Carrera de Nutrición y Dietética, Instituto de Investigación e Innovación en Salud Integral (ISAIN), Universidad Católica de Santiago de Guayaquil, Guayaquil, Ecuador; ^2^Carrera de Nutrición y Dietética, Facultad de Ciencias Médicas, Universidad de Guayaquil, Guayaquil, Ecuador; ^3^Departamento de Nutrición, Centro Médico Militar, Guatemala, Mexico; ^4^Carrera de Nutrición y Dietética, Facultad de Ciencias de la Vida, Escuela Superior Politécnica del Litoral, Guayaquil, Ecuador

**Keywords:** food insecurity, low food security, sarcopenic obesity, sarcopenia, older adults

## Abstract

Sarcopenic obesity is characterized by the loss of muscle strength, mass and muscle functionality and increased adipose tissue (obesity) according to different criteria and cut-off points. The prevalence of sarcopenic obesity among older adults is growing worldwide, and many factors are involved in its development. Diet and food security have been described as the main contributors to the development of obesity and sarcopenia. Food insecurity consists of limited or uncertain access to adequate and nutritious foods. This narrative review aims to summarize the existing data on food insecurity as a risk factor for sarcopenic obesity in the elderly.

## Introduction

It is widely recognized that aging and obesity are common public health issues worldwide. According to the World Population Prospect of the United Nations, in 2017, adults older than 65 involved almost 13% of the global population. This percentage is increasing at a more rapid rate compared to the portion of any other age group, and it is due to recent changes in the increase in life expectancy (1). Along with aging comes a loss of skeletal muscle mass and function, commonly accompanied by body fat (BF) gain.

Obesity is considered a worldwide pandemic (2), and is usually diagnosed by body mass index (BMI) above 30 kg/m^2^. Currently, some factors have been identified as contributors to obesity, such as genetic predisposition and unhealthy lifestyle, especially sedentary habits and excess of caloric intake (3).

Not only obesity is linked to metabolic syndrome, type 2 diabetes (T2D), and some cardiovascular events, as well as the development of drastic personal, social, economic, and healthcare tasks. Obese individuals are at higher risk of chronic and acute diseases, including end-stage organ failures, cancer, and infections, which can lead to complications and hospitalizations. Indeed, the rising rates of obesity have contributed to the coexistence of this disease with other comorbidities like sarcopenia (4).

The European Working Group on Sarcopenia in Older People (EWGSOP) in 2018 redefined the operational definition of sarcopenia as the presence of low muscle mass and strength, and severe sarcopenia if low physical performance is confirmed (5). Sarcopenia is considered as a syndrome with a multifactorial etiology whose prevalence increases with age (6). Several conditions in older adults have been described that may contribute to the development of sarcopenia, like lack of physical activity and dietary habits, which are similar to obesity-related risk factors (7). The term “sarcopenic obesity” (SO) was introduced to define a clinical and functional condition described by the conjunction of obesity and sarcopenia (6, 7).

Despite well-known risk factors for sarcopenia and obesity, growing evidence has shown that bad eating habits are one of these factors and can cause food insecurity. For example, in the United States, food insecurity increased significantly from 5.5 to 12.4% in the last ten years among older adults, and they have been related to obesity and sarcopenia (8–10). However, there is no scientific evidence exploring food insecurity as a risk factor for SO in aged people. For this reason, it is essential to describe how food insecurity may contribute to SO in older adults.

This narrative review aims to summarize the existing data on food insecurity as a risk factor for SO in the elderly. To accomplish this and contribute to the field, we will describe the presence of obesity and sarcopenia in older adults and the conjunction of both pathologies, food insecurity, and dietary intake as risk factors for SO.

## Materials and methods

A comprehensive search was carried out through PubMed and Web of Science for papers published until August 2022. We reviewed studies focusing on SO and its determinants, the relationship better obesity and food insecurity, and sarcopenia and food insecurity.

### Body composition changes in older adults

The aging process causes many changes in body composition (BC) of the elderly population, regardless of metabolic and physiological functions, resulting in a decrease in lean mass, also called fat-free mass (FFM) (11). Related to fat mass (FM), there is an increase in adiposity and a redistribution of fat from subcutaneous regions to intra-abdominal, intrahepatic, and intramuscular areas, characteristics associated with diseases such as diabetes and cardiovascular disease (12).

Older adults have more body fat percentages (BFP) than their younger counterparts, and there are gender differences in anthropometric measures and BFP. For example, women showed higher values of BMI, subcutaneous fat, and BFP; older men are prone to show more weight and lean mass (13).

Due to these BC changes, aging is linked with obesity and probably with sarcopenia or both of them. These conditions could aggravate disability and frailty, increasing morbidity and mortality rates (14).

Sarcopenia and Obesity have to be measured by using validated methods for measuring body composition, like whole-body Dual-energy X-ray Absorptiometry (DXA), bioelectrical impedance analysis (BIA), Computed Tomography (CT), and Magnetic Resonance Imaging (MRI) to diagnose obesity, sarcopenia, or both of them (5) DXA evaluates bone mineral density and is considered a method to assess whole-body and regional soft-tissue composition, which provides good data about the total amount of FM and FFM by body segments (13). BIA is an adequate and low cost method to measure total water and FFM. CT has accurate and reproducible data for FM, FFM, and visceral fat. MRI identifies changes in muscle structure (5). Using these methods, measurements of FM and FFM can properly make an accurate diagnosis against traditional anthropometric measurements.

### Obesity in older adults

The World Obesity Federation in 2017 stated that obesity is a chronic disease. There is a link between FM and the vulnerability of the host, making it significant as a health problem (15). There has been an increase of obesity at all ages on the past decade. In older adults, the prevalence of obesity on both sexes has been found at 37.5 and 39.4%, respectively (16). The association between excess of body fat in older adults and the whole or specific disease mortality is still under debate (17). Obesity is defined, regardless of age, as a BMI greater than or equal to 30 kg/m^2^. There is no consensus regarding the best measure of obesity in the older population. BMI is an easy tool that correlates with the percentage of body fat in young and middle-aged adults. However, physiological changes in BC render BMI less accurate with aging.

The changes in age-related BC, specially fat distribution, could assist in perceiving the link between adiposity, morbidity, and mortality in older adults. Recent data suggest that the distribution of FM, visceral fat, and reduction of FFM would be more relevant than BMI seeking health problems associated with obesity in older adults (17).

In 2007, the Spanish Society of Parenteral and Enteral Nutrition published the following BMI cohort points to identify overweight and obesity in older adults: 27.0–29.9 kg/m^2^ (overweight); 30.0-34.9 kg/m^2^ (obesity class I); 35.0–39.9 kg/m^2^ (obesity class II); 40.0–49.9 kg/m^2^ (obesity class II); and 50 kg/m^2^ (obesity class IV) (18).

### Sarcopenia in older adults

Sarcopenia is a progressive and generalized skeletal muscle disturbance (5). The prevalence range may differ depending on the clinical scenario, and the main difference can be the method proposed for the description. Peterman-Rocha et al. found that the prevalence of sarcopenia varies significantly in the systematic (10–27%) vs. a narrative review (0.2–86.5%), regardless of the differences in the methodology and cut-off points (19). These differences can be due to the scientific criteria of the selection of the studies. The classification systems most usually used are EWGSPOP and the Asian Working Group on Sarcopenia (AWSP) (19). In 2019, Rodriguez-Rejón et al. found that despite the methodology used to diagnose sarcopenia, the results did not change in frequency (20).

Primary sarcopenia occurs with aging, and secondary sarcopenia develops due to physical inactivity, malnutrition, and diseases, such as neurodegenerative disease, endocrine disease, or malignancies (5).

This condition is associated with increased adverse outcomes, including falls, functional decline, frailty, and mortality. At first, sarcopenia was an age-related process in older people (21); nowadays, evidence shows it can be present across the lifespan and is influenced by lifestyle risk factors (22), genetics, and also secondary to disease (23).

In order to assess sarcopenia, revised guidelines suggest measuring muscle functionality and validating strength, which is better at predicting unfavorable outcomes (24). Actually, EWGSOP2 valued low muscle strength as a leading parameter of sarcopenia and an adequate measure of muscle function. However, sarcopenia is probably when low muscle strength is present. The diagnosis is made when the low muscle quantity is registered, and severe sarcopenia when quality is confirmed (5).

### Sarcopenic obesity

Sarcopenic obesity (SO) was first described by Baumgartner in 2000 as a clinical and functional condition characterized by the presence of obesity and the diagnosis of sarcopenia (7, 25, 26). Both conditions, loss of MM and muscle capability and gaining body fat, may increase the risk for non-communicable diseases like diabetes and cardiovascular disease and increase the chances of adverse health outcomes such as disability or impairment, cardiometabolic diseases, other comorbidities, and mortality more than sarcopenia or obesity individually (5, 26).

Sarcopenia and obesity share pathological factors, including aging, changes in BC, inflammation, and hormones (27). The aging process carries a low metabolic rate and metabolic adaptations, including adaptive thermogenesis and changes in oxidative capacity; this process favors the development and onset of SO (28). Changes in BC related to age are the main risk factor for SO. Studies have shown that FM increases with age, especially between 60 and 75 years old (27–29). Muscle mass and strength decline progressively around 30, accelerating after 60 years. In addition, there are changes in fat accumulation with aging; visceral fat and intramuscular fat tend to increase, while subcutaneous fat in other body regions declines. Fat infiltration is associated with lower muscle strength and leg performance capacity (29).

Another associated risk is low physical activity, a well-known risk factor of obesity, low muscle strength, muscle atrophy, and reduced metabolic rate (30).

Inflammation also contributes to SO (31). The adipose tissue produces pro-inflammatory cytokines such as interleukin-6, tumor necrosis factor-alpha, and adipokines such as leptin and adiponectin, which regulate the inflammatory response (32, 33). Obese subjects have a pro-inflammatory state which may be one of the key factors in decreasing muscle strength, creating a vicious cycle (33).

Obese individuals have muscle catabolism because insulin has no anabolic function due to insulin resistance (34). Insulin resistance correlates independently with poor muscle strength, and older patients with diabetes show accelerated loss of leg muscle strength and quality (34). Indeed, low levels of sex-specific hormones are an important factor related to SO (34). After menopause, a decline in estrogen levels can result in increased body weight and FM as well as shifts in the accumulation of fat from subcutaneous to visceral deposits. In older men, total lower levels of testosterone are associated with sarcopenia and may contribute to muscle impairment in obese individuals (35, 36).

On the other hand, obesity can independently lead to loss of muscle mass and function due to the negative impact of adipose tissue, which causes metabolic alterations that include oxidative stress, inflammation, and insulin resistance. All of them negatively affect muscle mass (37).

In addition, diet is a risk factor that affects sarcopenia and obesity by different mechanisms. Generally, sarcopenia is associated with an insufficient nutritional intake, whereas obesity is an outcome of excessive energy intake, leading to an imbalance between energy intake and energy expenditure (38).

Due to the clinical and epidemiologic importance of assessing SO, screening tools and diagnostic criteria for this condition have been published recently (5). However, despite the classification, the method used for screening and diagnosing sarcopenia is critical for appropriate and prompt interventions in older adults.

### Screening and diagnosis of sarcopenic obesity

Screening of SO is based on elevated BMI or waist circumference and indicators of sarcopenia such as clinical symptoms and risk factors or the use of SARC-F. The clinical symptoms and risk factors include age >65 years, chronic diseases, recent acute disease or nutritional alterations, repeated falls, weakness, exhaustion, fatigue, and movement limitations (Table 1) (6).

**TABLE 1:** Screening, diagnosis, and staging of sarcopenic obesity.

Screening of SO	• High BMI and WC • Clinical symptoms, clinical suspicion SARC-F • If conditions present, go to Diagnosis
Diagnosis of SO	• HGS, Chair stand test if positive, go to body composition. • Increased Fat mass, reduced muscle mass using medical imaging, BIA, calf circumference • Items 1 and 2 must be present.
Staging of SO	Based on the presence of complications resulting from high FM and low ASMM • Stage I: No complications • Stage II: presence of at least one complication attributable to SO: metabolic diseases, functional disabilities, cardiorespiratory diseases,

SO, sarcopenic obesity; BMI, body weight index; WC, waist circumference; HGS, hand grip strength; BIA, bioelectrical impedance analysis; FM, fat mass; ASMM, appendicular skeletal muscle mass. Adapted from the Definition and Diagnostic Criteria for Sarcopenic Obesity: ESPEN and EASO Consensus Statement (6).

To diagnose sarcopenia and obesity, MM and FM body can be assessed by diverse techniques, such as anthropometry, BIA, and medical imaging. Anthropometry includes BMI diagnosis, skin-fold thickness, and body circumferences. Despite some studies of cut-off points for older adults, the limitation is the lack of precision in evaluating the MM and the high probability of error (39, 40).

BIA measures MM and FM established on electrical conduction through tissues; limitations include hydration levels, exercise, and food or liquid intake. Medical imaging has an advantage for diagnosing and grading sarcopenia and obesity. It is an accurate and reliable method and can be applied for longitudinal changes in clinical trials and as a treatment assessment tool (41).

SO, as other clinical conditions, require standardization and cut-off points for diverse populations or ethnicity, especially due to dissimilarity regardless of body type, adiposity, and lifestyles of the worldwide population, mainly for the Asian and Latin American populations.

Diagnosis of SO should include altered skeletal muscle functional parameters and altered BC (42). For skeletal muscle functional parameters, hand grip strength and knee strength can be used (43). For BC, DEXA or BIA are recommended, and if possible, TC (44).

The staging of SO should be based on the presence or absence of complications. The screening, diagnosis, and staging summary are presented in Table 1, adapted from the Definition and Diagnostic Criteria for Sarcopenic Obesity: ESPEN and EASO Consensus Statement (6).

### Food insecurity

Food insecurity is a severe global public health concern. Around 2,300 million people worldwide were affected by moderate or severe food insecurity in 2021 due mainly to conflicts, meteorological phenomena, and socio-economic perturbations (45). There should be an update of food and agricultural policies to make healthy diets more affordable. Food insecurity is defined as limited or uncertain access to adequate and nutritious foods due to many factors, especially financial resources (46).

Food insecurity and hunger are concepts that could seem like synonyms. Nevertheless, food insecurity is a household-level economic and social condition limiting access to food, and hunger is an individual-level physiological condition resulting from food insecurity (47). In addition, The United States Department of Agriculture (USDA) classifies food insecurity as reduced quality, variety, or desirability of the diet with little or no indication of reduced food intake and very low food security as multiple indications of disrupted eating patterns and reduced food intake (48).

It has been reported that food insecurity increases have affected people of all ages, particularly those in vulnerable situations (48). Older adults are exposed to many conditions that increase their vulnerability. Food insecurity has been well described as a critical factor that leads to health issues in the aged population, mainly affecting those primarily alone, who have fixed incomes and chronic health concerns (49, 50). Consequently, the prevalence of chronic diseases, poor management of chronic diseases, and decreased health-related quality of life in older adults are associated with food insecurity (49, 51).

One of the main ways that food insecurity can be a determinant of health and diseases is its impact on diet quality.

### Food insecurity and diet quality

Diet quality (DQ) can be determined by many indicators such as specific nutrient quantitative and qualitative content or by designed tools that assess an individual’s overall diet quality (52, 53).

Poor DQ is a direct and preventable cause of death globally. Food insecurity has been associated with lower DQ (54), particularly with more than 50 adverse associations with differences according to sex and ethnicity (55, 56).

The decline in diet quality has been observed in cohorts that aged from middle to older adulthood, especially in response to drastic changes derived from social or health issues (57, 58). The proportion of US older adults with poor diet quality significantly increased from 50.9 to 60.9% between 2001 to 2018, with a significant decreasing trend in diet scores among both sexes and all age groups (59).

DQ can also impact nutritional status. Carrier et al. found that diet quality was associated with malnutrition in older adults living in long-term care. In this cohort, several individual nutrients were associated with low calf circumferences (<31 cm). In the same study, older adults with better dietary quality and habits were more likely to have better nutritional status. In addition, a US longitudinal study showed that malnutrition was significantly associated with poorer diet quality and lower energy and protein intake (60).

It is important to mention that sarcopenia could increase the risk of inadequate diets. For example, in long-term care homes in Spain, the risk of a poorer diet was higher in females and residents with sarcopenia (61). In community-dwelling older adults, diet quality has been associated with the number of comorbidities and baseline risk of malnutrition (62). In contrast, prospective associations of poor DQ with long-term incidence of protein-energy malnutrition have not been found (63).

Those differences could result from the method to assess DQ, the definition of malnutrition or risk of malnutrition, and institutional or community-dwelling older adults’ location.

On the contrary, a recent systematic review and meta-analysis describe a significant association between healthy dietary patterns and maintenance of gait speed with age, an indicator of sarcopenia risk (64).

### Food insecurity and obesity in older adults

Obesity and food insecurity are both public health concerns influenced by social disparities that impact the quality of life in older adults (64). Social and economic transitions in low and middle-income countries are contributing to an increase in the aging population and, together with the added burden of poverty and inequities, increase food insecurity, obesity, and associated comorbidities (65). Food insecurity is paradoxically associated with obesity in high-income countries. The first hypothesis of whether food insecurity causes obesity was published 27 years ago (66).

Explaining the link between food insecurity and body weight is a complex issue to the fact that it is not well understood what mechanisms cause it. One of the most studied hypotheses describes that food insecurity can cause obesity due to the high calorie and palatable food consumed by low food secure populations such as older adults could be (67). Also, the food insecure population could have limited knowledge about nutrition and resources to follow a healthy lifestyle, so they might have fewer opportunities to keep healthy eating and exercise to prevent and treat obesity (68). Furthermore, older adults’ nutrition knowledge can influence positively or negatively their health status and quality of life (69).

The resource scarcity hypothesis suggests that perceived food insecurity in a permissive environment with access to high-calorie foods may cause positive energy balance in individuals of low social status or socially vulnerable populations (70).

Physical limitations can also be a potential risk factor for food insecurity and the food insecurity-obesity paradox in older adults (71). However, some hypotheses suggest a bidirectional association between food insecurity and physical limitations (72).

In particular, the association between food insecurity and obesity has shown mixed findings by age and gender. This association appears not to be present in older men even though food insecurity and obesity coexist among low-income, older women due to differences in household income, educational attainment, and social networks (73–77).

### Food insecurity and sarcopenia in older adults

**FIGURE 1:**
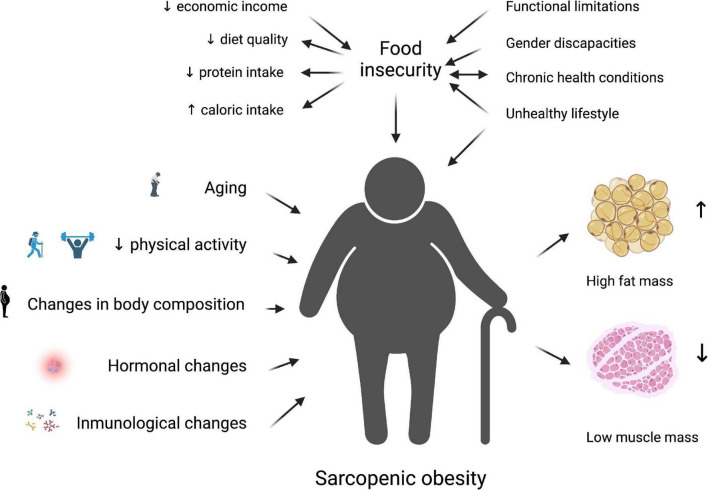
The interrelation between food insecurity and sarcopenic obesity in older adults is related to low economic income, impacting a low-quality diet with a small protein intake with a high energy offer. This scenario is worsened by unhealthy lifestyles and the presence of comorbidities, and body composition alteration due to hormonal and immunological changes, resulting in low muscle mass and high-fat mass. This image was created using Biorender.com.

Not long ago, it was found that individuals who experienced food insecurity had lower muscle mass strength and physical performance (78, 79). On the contrary, functional limitations are significantly associated with increasing food insecurity in older adults, and these associations could be influenced by ethnicity (80). Currently, food insecurity is strongly associated with sarcopenia (81–84). Prevalence of sarcopenia in older adults with food insecurity has been described in 24,4% in low- and middle-income countries; indeed, severe food insecurity was associated with 2.05 times higher odds for sarcopenia (84). Figure 1 illustrates the previously described interaction between food insecurity and SO in older adults.

The double burden of malnutrition has been proposed as a term that can describe a scenery where critical nutrients are poor independently of excess energy intake. In this sense, the excess FM can lead to sarcopenia and, consequently, SO (85).

Against these conditions, improve food insecurity (86), healthy eating patterns such as the Mediterranean, and physical activity (87), have been described as strategies to achieve healthy aging and reduce the risk of obesity and sarcopenia.

## Conclusion

Sarcopenia and obesity share pathological factors, including aging and changes in BC. Changes in BC are the leading risk factor for SO. That is why SO needs to be screened and diagnosed adequately. The relationship between food insecurity and SO is mediated mainly by diet and disabilities associated with aging. Food insecurity can determine diet quality which is an important modifiable risk factor in the development of sarcopenia and obesity.

## Author contributions

All authors have conceptualized this narrative review, analyzed current literature, wrote the original draft, reviewed the final version, and agreed to the published version of this manuscript.
